# Chronic PD-1 Checkpoint Blockade Does Not Affect Cognition or Promote Tau Clearance in a Tauopathy Mouse Model

**DOI:** 10.3389/fnagi.2019.00377

**Published:** 2020-01-14

**Authors:** Yan Lin, Hameetha B. Rajamohamedsait, Leslie A. Sandusky-Beltran, Begona Gamallo-Lana, Adam Mar, Einar M. Sigurdsson

**Affiliations:** ^1^Department of Neuroscience and Physiology, Neuroscience Institute, New York University School of Medicine, New York, NY, United States; ^2^Department of Psychiatry, New York University School of Medicine, New York, NY, United States

**Keywords:** tau, Alzheimer’s disease, tauopathy, PD-1 blockade, antibody, therapy, mouse models, behavior

## Abstract

Programmed cell death protein 1 (PD-1) checkpoint blockade with an antibody has been shown to reduce amyloid-β plaques, associated pathologies and cognitive impairment in mouse models. More recently, this approach has shown effectiveness in a tauopathy mouse model to improve cognition and reduce tau lesions. Follow-up studies by other laboratories did not see similar benefits of this type of therapy in other amyloid-β plaque models. Here, we report a modest increase in locomotor activity but no effect on cognition or tau pathology, in a different more commonly used tauopathy model following a weekly treatment for 12 weeks with the same PD-1 antibody and isotype control as in the original Aβ- and tau-targeting studies. These findings indicate that further research is needed before clinical trials based on PD-1 checkpoint immune blockage are devised for tauopathies.

## Introduction

Programmed death ligand 1 (PD-L1) binds to its receptor, programmed cell death protein 1 (PD-1), resulting in complex effects on the immune system (Chamoto et al., 2017; Wei et al., 2018). PD-1 and PD-L1 inhibitors block this association and are primarily used today to treat cancer, in which interaction of PD-L1 on tumor cells with PD-1 on T-cells reduces T-cell function and thereby prevents the immune system from attacking the tumor cells (Chamoto et al., 2017; Wei et al., 2018).

In 2016, it was reported that PD-1 immune checkpoint blockage cleared amyloid-β (Aβ) in two mouse models following a few antibody injections at ages when Aβ plaques are abundant (Baruch et al., 2016). Cognition was assessed in one of the models and was improved as well. The putative mechanism put forward was that PD-1 blockage led to the recruitment of monocyte-derived macrophages to the brain, which then cleared Aβ resulting in improved cognition. Following these interesting findings, several pharmaceutical companies that were developing PD-1 antibody blockers for other conditions pursued this approach with their own compounds and in different Aβ plaque mouse models. In their hands, PD-1 immunotherapy stimulated systemic activation of the peripheral immune system as expected, but monocyte-derived macrophage infiltration into the brain was not detected, and brain Aβ pathology was not altered (Latta-Mahieu et al., 2018).

Recently, the same group that showed beneficial effects of PD-1 blockade on Aβ clearance and cognition followed up on their pioneering findings demonstrating that similar benefits could be obtained in a tauopathy model using the same PD-1 antibody and isotype control as in their original study (Rosenzweig et al., 2019). Comparable results were observed with a PD-L1 antibody. We have examined this approach in a different more commonly used tauopathy model, intervening also at a stage when tau pathology is clearly evident. Twelve weekly injections of the same PD-1 antibody as used in both of the effective Aβ and tau-targeting studies, slightly increased locomotor activity but failed to significantly reduce tau pathology or affect cognitive performance in five different tasks.

## Materials and Methods

### Mice

Homozygous female JNPL3 tauopathy mice of a mixed background were used for this study (Taconic, Germantown, NY, USA, Stock # 2508), starting at 10–11 months of age (*n* = 22: 11 treated and 11 controls) and concluding at 14–15 months following extensive behavioral testing (*n* = 18 brains analyzed: 10 treated and eight controls). Six mice died during the study, four controls and two treated. Two controls died after the first and eighth injection, respectively. One mouse in each group was euthanized before the Barnes maze. Those two brains were saved and included in the analyses. In addition, one mouse in each group died before the fear conditioning test. This mouse model expresses ubiquitously the human P301L tau mutation within the 0N4R isoform under the direction of a prion promoter and is one of the most widely used tauopathy mouse models. The homozygous animals have an early onset of tau pathology, which is most severe in the brainstem and spinal cord but is evident throughout the brain. In the original description of this model, the motor phenotype was severe with progressive hindleg paralysis leading the animals to not being able to ambulate or feed themselves properly (Lewis et al., 2000). This was more severe in females and in homozygous animals, with functional deficits starting at about 5 months and not many females survived past 1 year of age. Because of the motor phenotype, cognitive tests were not commonly employed in this model in the first years after it was developed because those typically require intact motor functions. Currently, the tau pathology and related motor deficits have shifted to an older age in this model, with motor deficits starting at 8–12 months and most animals survive well into their second year of age, in particular the males, with females showing more severe pathology (EM Sigurdsson, personal observation). Because of sex differences in pathology, only females were used in the present study and all appeared healthy at the onset of the study. The mice were housed in Association for Assessment and Accreditation of Laboratory Animal Care (AAALAC) approved facilities. All mouse care and experimental procedures were compliant with guidelines of animal experimentation and were approved by the Institutional Animal Care and Use Committee at New York University School of Medicine.

### Antibody Administration

PD-1 checkpoint inhibitor (anti m PD-1; Rat IgG2a isotype, clone RPM1-14, Bioxcell Catalog# BE0146) or its isotype control (rat IgG2a, clone 2A3 Bioxcell Catalog# BE0089) were administered intraperitoneally at a dose of 10 mg/kg every week for 12 weeks. The PD-1 antibody blocks PD-1 and PD-L1 binding and boosts the immune response. The mice went through sensorimotor tests following the 12th injection at 13–14 months of age, followed by cognitive testing. The brains were subsequently harvested at 14–15 months of age.

### Behavior

Each instrument was wiped down with 30% ethanol and allowed to completely dry between each mouse tested. Before each testing session, the mice were acclimated to the testing room conditions for a minimum of 30 min.

### Sensorimotor Tests

#### Locomotor Activity

A circular open field activity chamber (56 cm in diameter, 31 cm in height) was used to measure exploratory locomotor activity over 15 min as we have routinely assessed previously in this model (Asuni et al., 2007; Boutajangout et al., 2011). A camera placed above the field recorded animal movements (Ethovision XT, Noldus, Leesburg, VA, USA). Measured parameters were distance traveled (cm) and mean velocity (cm/s).

#### Rotarod

This test was conducted to measure limb motor coordination and balance (San Diego Instruments, San Diego, CA, USA). Initially, the mice were habituated to the instrument and the task by a four trial training session to obtain a baseline level of performance. Each training trial consisted of 5 min, with 15 min break between sessions, and the speed of the rotating rod increased linearly from 0 to 50 revolutions per min (rpm) during the 5 min period. The next day, the mice went through four trials with 15 min break between sessions. Each testing trial consisted of 10 min, with the speed of the rod increasing linearly from 0 to 40 rpm during that period until the mouse fell off or inverted (by clinging) from the rotating rod. The distance traveled and latency at that point were recorded. To prevent injury, a soft foam cushion was placed beneath the rod.

### Cognitive Tests

#### Object Recognition Test

The spontaneous object recognition test (ORT) that was used measures deficits in short-term memory, and was conducted in the circular open field activity arena mentioned above (56 cm in diameter). A video camera mounted above the arena automatically recorded mouse movement and time spent by the objects and in different zones. At the training session, two identical novel objects were placed in opposite quadrants of the arena, and the mouse was allowed to explore for 15 min. For any given trial, the objects in a pair were 10 cm high and composed of the same material so that they could not readily be distinguished by olfactory cues. The time spent exploring each object was recorded by a tracking system (Ethovision XT, Noldus), and at the end of the training phase, the mouse was removed from the arena for the duration of the retention delay (3 h). Normal mice remember a specific object after a delay of 3 h and spend the majority of their time investigating the novel object during the retention trial. During the retention test, the animals were placed back into the same arena, in which one of the previous familiar objects used during training was replaced by a novel object, and allowed to explore freely for 6 min. The time spent exploring the novel and familiar objects was recorded for the 6 min period. The percentage short-term memory score is the time spent exploring the novel object compared to the old object during the retention session. Typically, an animal with intact memory spends about 70% of the time with the novel object. For the long-term memory test, the mice were replaced back into the same arena 24 h after the short-term test, with a different novel object used, whereas the familiar object was the same as before. For both tests, each mouse was exposed to the same familiar and novel objects but their location was counterbalanced across animals within each treatment condition.

#### Spontaneous Alternation Test

The spontaneous alternation test was conducted in a Y-maze apparatus. The Y-maze apparatus (Med Associates) was comprised of a central hub (working area: 8.3 cm L × 8.3 cm W × 12.7 cm H) attached to three arms (working area: 36 cm L × 7.3 cm W × 12.7 cm H) equally spaced 120° apart. Clear polycarbonate walls and removable ventilated lids ensured that mice were confined to the maze but had access to all surrounding visual cues in the testing room.

The test consisted of a single 5 min trial in which the mouse was allowed to freely explore all three arms of the Y-maze. Mice were started in the central hub facing a wall to help avoid placement or initial arm bias. Mice were tracked by an overhead camera and video acquired and analyzed using Noldus Ethovision software (v11.5). Locomotor activity was assessed using metrics of the total number of arm entries (from the central hub) and the total distance traveled. Spontaneous Alternation [%] was defined as consecutive entries in three different arms (ABC), divided by the number of possible alternations [total arm entries minus 2; (Sarter et al., 1988)]. Re-entries into the same arm were rated as separate entries, which resulted in a 22.2% chance level for continuous alternation.

#### Novel Location Test

The Novel Location test was conducted in the same Y-maze apparatus described above. To maximize novelty, a different set of arms was used and the maze configuration was rotated by 45° relative to the position for the Spontaneous Alternation test so that the vantage of distal cues would differ between tests. Mice were tracked by an overhead camera and video acquired and analyzed using Noldus Ethovision software (v11.5).

The novel location test consisted of two 3 min trials. In trial 1, each mouse was started in the central hub facing a wall. The mouse was permitted to explore two arms of the Y-maze, while entry into the third arm was blocked by an opaque acrylic door. The location of the blocked arm was randomized across mice. After trial 1, the mouse was briefly returned to its home cage and the maze was cleaned with 30% ethanol and left to dry completely. The inter-trial interval (ITI) was 1 h. In trial 2, the door in front of the blocked arm was removed and the mouse was again placed into the central hub facing a wall and then allowed to access all three arms of the maze. Locomotor activity was assessed using metrics of the total number of arm entries (from the central hub) and the total distance traveled. Time in Novel Arm [%] was defined as the time spent in the novel arm divided by the time spent in all arms during the 3 min trial. The chance performance on this measure is 33.3%.

#### Barnes Maze

The Barnes Maze procedure was designed to provide an index of visuospatial learning and memory. The test comprises a series of trials in which rodents learn to navigate to a hole box location providing shelter from a noisy, brightly-lit platform (Barnes, 1979). The protocol we implemented was adapted based on a hybrid of prior studies suggesting optimal parameters for platform characteristics (O’Leary and Brown, 2013), as well as for the number and spacing of training and probe trials (Sunyer et al., 2007; Illouz et al., 2016). Our previous experience using these parameters has produced reliable spatial learning in wild type mice, based primarily on the use of extra-maze cues.

The maze apparatus consists of a beige, textured plastic platform surface, 36” in diameter, elevated 36” from the floor on a black-painted wooden stand (San Diego Instruments, San Diego, CA, USA). The platform has 20 2” holes equally spaced around the periphery of the platform (~1” from the edge). For a given subject, a gray plastic escape box (~10 cm × 8.5 cm × 4 cm) was located under one of the holes that were kept consistent across trials. Visible distal cues were placed around the room and remained constant throughout the testing period. The maze was illuminated by an overhead lamp (100W) placed 30” above the center of the platform. Behavior was recorded using an overhead camera for later tracking and analysis using Noldus Ethovision software (v11.5).

On the first day, mice were given two trials: a guided, habituation trial and a standard escape trial. For the habituation trial, each mouse was gently removed from their home cage by the base of their tail and placed in the center of the platform under an inverted, transparent 500 ml beaker for 1 min. A white noise generator (~90–100 dB at platform level) was turned on when the mouse was placed onto the maze. After the 1 min period, the beaker was slowly moved with the mouse inside to guide them to the target hole. The mouse was then allowed or was gently guided by the experimenter using the beaker, to enter the escape box. Once inside, the mouse was permitted 2 min to explore the escape box before being transported back to its home cage within the box. On both the habituation and standard escape trials, the white noise was turned off as soon as the mouse entered the escape box. Between each training trial, the maze was rotated such that the hole positions were maintained relative to the room and distal cues. This was done to ensure that only distal spatial cues (not local or non-spatial landmark cues) could be used to guide successful escape navigation. The target hole was counterbalanced across animals within each treatment condition.

After the initial beaker trial (2 h ITI), each mouse was given a standard escape trial in which the mouse was gently placed onto the center of the maze within a plastic 6” (L) × 6” (W) × 8” (H) start box. The white noise was turned on and, after 10–15 s, the start box was lifted. The mouse was given a maximum of 3 min to find the designated hole and escape box. When a mouse entered the escape box, the white noise was turned off and the animal was given 1 min inside the box before being transferred back to the home cage. If the mouse did not find the escape hole within the 3 min limit, it was slowly guided to the target hole under the inverted beaker (as described above), and given 1 min inside the box (with white noise turned off) before being transferred back to the home cage.

After the first two trials on day 1, each mouse was given three standard escape trials per day—with the maze rotated after each trial—over the next three consecutive days.

The day following the 10th standard escape trial, a probe trial was conducted in which the escape box was removed to help control for possible strategies that used local cues from the box, This trial was started similar to training but was only 120 s in duration. Latency, distance and the number of mistakes (non-target holes visited) until finding the target hole as well as average proximity to the target hole on the probe trial were recorded as indices of spatial memory.

#### Fear Conditioning

Fear conditioning is an associative learning task in which mice are presented with a behaviorally neutral conditioning stimulus (CS), such as an auditory stimulus, that is presented in temporal proximity with a mild foot shock that acts as an aversive unconditioned stimulus (US; Maren, 2001). In the task, the mice learn that the CS predicts the US and will subsequently elicit specific behavioral responses such as freezing when the CS is presented on its own. Mice additionally learn to associate the environment or context of the chamber in which the CS-US pairings take place with the US, and will elicit specific behavioral responses when in the environment/context in the absence of the CS.

The fear conditioning apparatus consists of four identical square-shaped chambers (Coulbourn Instruments). The floor of the chamber has a metal grid through which the shock US is delivered, and the walls of the testing chamber are made of aluminum and clear acrylic. All chambers are mounted within specially designed light and sound attenuating outer boxes ventilated by a small fan. To aid in establishing a context, chambers were scented with three drops of mild natural extract (vanilla or lilac) added to a gauze inside a small plastic dish placed within the waste pan below the grid floor and out of reach of the subject. Each chamber was illuminated by small white, blue and/or infrared LEDs to provide low-ambient light, with speakers mounted on one wall and a camera mounted overhead to record video of behaviors during the training and testing sessions. Chamber, scent and lighting conditions were counterbalanced across the drug testing groups.

The fear conditioning protocol comprised four phases: (1) a conditioning phase in which mice receive CS-US context/tone-shock pairings; (2) a short term (1 h post-conditioning); (3) a long term (24 h postconditioning) testing phase in which the extent of associative fear memory for the context is assessed by examining behavior during re-exposure to the same testing chambers (without tone CS or US shocks); and (4) a testing phase for associative fear memory for the auditory white noise cue in different chamber context (with tone CS but no shocks 48 h post-conditioning). The conditioning phase comprised one 6 min session, in which each mouse was placed alone into a specified chamber and given a 2 min acclimation period in which no stimuli or shocks were delivered. Then animals were given two noise-shock pairings, each of which comprised a 30 s auditory white noise CS (78 dB) presented prior to delivery of 2 s 0.2 mA shock current US *via* a scrambled signal across the stainless steel grid floor overlapping with the last 2 s of the noise stimulus. Spacing between the noise-shock CS-US pairing was 2 min (each pairing initiated at 3 min and 5 min within the trial). After the second shock, animals were left in the chamber another 30 s prior to being gently removed from the chamber and placed back into their home cage.

Associative learning and memory were evaluated in the subsequent three sessions. To assess short-term and long-term associative fear memory for the context, mice were gently placed into the same chamber used to assess the extent of contextual learning for 5 min at 1 h and 24 h time points after the conditioning session, respectively. To assess associative fear conditioning for the white noise cue, a 20 min session was given in a novel chamber context 48 h after the conditioning session. The white noise stimulus was presented alone for 3 min after 3 min within the chamber and then a 30 s white noise was played and an additional six times at 3 min intervals. After each session, mice were gently removed from the chamber and placed back into their home cage.

Associative learning was determined by the amount of time the mice spend freezing or immobile during these sessions, in the presence or absence of the white noise. Percent freezing was extracted using the automated Freeze Frame 3 software (threshold = 10, duration criterion = 2 s).

### Western Blotting

The left hemisphere brain tissue was homogenized in (5× vol/w) modified radioimmunoprecipitation assay (RIPA) buffer (50 mM Tris-HCl, 150 mM NaCl, 1 mM ethylene diamine tetra-acetic acid (EDTA), 1% Nonidet P-40, pH 7.4) containing protease and phosphatase inhibitors (4 nM phenylmethylsulfonyl fluoride (PMSF), 1 mM NaF, 1 mM Na_3_VO_4_, 1× Complete protease inhibitor cocktail (Roche, Indianapolis, IN, USA), and 0.25% sodium deoxycholate). The homogenate was then centrifuged (20,000× *g*) for 20 min at 4°C and the supernatant collected as soluble tau fraction [low-speed supernatant (LSS)]. To obtain the sarkosyl insoluble tau fraction, equal amounts of protein from the LSS were mixed with 10% sarkosyl solution to a final 1% sarkosyl concentration and incubated on a rotator for 30 min at room temperature. The samples were then centrifuged at 100,000× *g* in a Beckman TL-100 ultracentrifuge at 20°C for 1 h. The pellet was resuspended in 100 μl 1% sarkosyl solution and spun again at 100,000× g at 20°C for 1 h. The supernatant was then discarded and the pellet air-dried for 30 min. Then, 50 μl of modified O+ buffer (62.5 mM Tris-HCl, 10% glycerol, 5% β-mercaptoethanol, 2.3% SDS, 1 mM EDTA, 1 mM ethylene glycol-bis(β-aminoethyl ether)-tetraacetic acid (EGTA), 1 mM NaF, 1 mM Na_3_VO_4_, 1 nM PMSF and 1× Complete protease inhibitor cocktail, including about 1 μg/ml of bromophenol blue) was added and the sample vortexed for 1 min, and then boiled for 5 min [Sarkosyl Pellet (SP) fraction] and kept frozen at −80°C until used for Westerns. The LSS fraction (500 μg—about 30 μl) was diluted with the modified RIPA buffer (about 220 μl) and modified O+ buffer (50 μl), resulting in a final protein concentration of 25 μg/15 μl, and processed in the same way (boiled for 5 min and frozen). For Western blots, the soluble and insoluble tau fractions were thawed, reboiled for 5 min and electrophoresed (15 μl per lane) on a 12% (w/v) polyacrylamide gel. The proteins were then transferred to a nitrocellulose membrane that was subsequently blocked in 5% nonfat milk with 0.1% Tween-20 in TBS for 1 h, and incubated with different antibodies at 4°C overnight (Tau-5 (SantaCruz, sc-58860, 1:1,000, tau epitope 210–241), PHF1 (1:1,000, tau epitope around aa P-Ser396) and CP27 (1:500, human tau specific epitope aa 130–150) generously provided as cell culture supernatants by Peter Davies). Following washes, the membranes were then incubated for 2 h with 1:2,000 horseradish-peroxidase (HRP) conjugated goat anti-mouse antibody (ThermoFisher Scientific), developed in ECL (ThermoFisher Scientific), imaged with Fuji LAS-4000, and the signal quantified with by ImageQuant software. All the samples within each figure panel were run on the same gel, and each set of blots was repeated at least twice with one set used for quantitation.

### Tau Sandwich Enzyme-Linked Immunosorbent Assay (ELISA)

For assessing human tau levels, a Corning Costar^®^ Assay Plate, 96 well Clear Flat Bottom Half Area High Binding Polystyrene (Ref 3690), was coated (50 μl/well) with the anti-human tau monoclonal antibody BT2 (1 μg/ml, ThermoFisher Scientific, MN1010, epitope aa 194–198) for 72 h at 4°C on a shaker. The plate was washed four times with wash buffer and then blocked with SuperBlock Blocking Buffer in TBS (ThermoFisher Scientific) for 90 min at room temperature on a shaker. Low-speed supernatant (LSS) samples (soluble tau) were first normalized to 2 mg/ml total protein concentration and then diluted 1:100 using RIPA base buffer. SP samples (insoluble tau) were diluted 1:100 using an O+ base buffer. Human recombinant tau standards [Tau-441 (2N4R), rPeptide], LSS, and SP samples were loaded in duplicate (20 μl/well) and then diluted 1:5 with 20% Super Block in 1× TBS in the plate and incubated overnight at 4°C on a shaker. The following day, the plate was washed four times with wash buffer and 0.2 μg/ml anti-human tau monoclonal antibody, biotin-labeled (HT7B; ThermoFisher Scientific, MN1000B, epitope aa 159–163) in 20% Super Block in 1× TBS was added to the plate (50 μl/well) and incubated at 37°C for 90 min. The plate was then washed four times with wash buffer and then Streptavidin Poly-HRP50 Conjugate (1:6,000 in 20% Super Block in 1× TBS, Fitzgerald Industries International, 65R-S10PHRP) was added (50 μl/well) and incubated in the dark at room temperature for 40 min on a shaker. The plate was then washed four times with wash buffer and then developed with 3,3′,5,5′-Tetramethylbenzidine Liquid Substrate, Super Slow, for enzyme-linked immunosorbent assay (ELISA; 50 μl/well) and the absorbance was read at 650 nm using a BioTek Synergy 2 plate reader.

### Data Analysis

All the data were plotted and analyzed using GraphPad 8.0. Normality was assessed with the D’Agostino-Pearson test. Data that passed the test, were analyzed with an unpaired *t*-test, and data that failed the test were analyzed with the Mann–Whitney test. Both tests were two-tailed. A *P*-value of less than 0.05 was considered significant.

## Results

Following weekly treatment with PD-1 antibody or isotype control IgG for 12 weeks, starting at 10–11 months of age, the homozygous JNPL3 mice underwent a battery of behavioral tests as outlined below, and in that particular order. Subsequently, their brains were analyzed for tau and phospho-tau levels in soluble and sarkosyl insoluble fractions by Western blots and ELISA to determine if the PD-1 treatment led to clearance of pathological tau protein.

### Behavior

#### Sensorimotor Tests

##### Locomotor Activity

**Figure 1:**
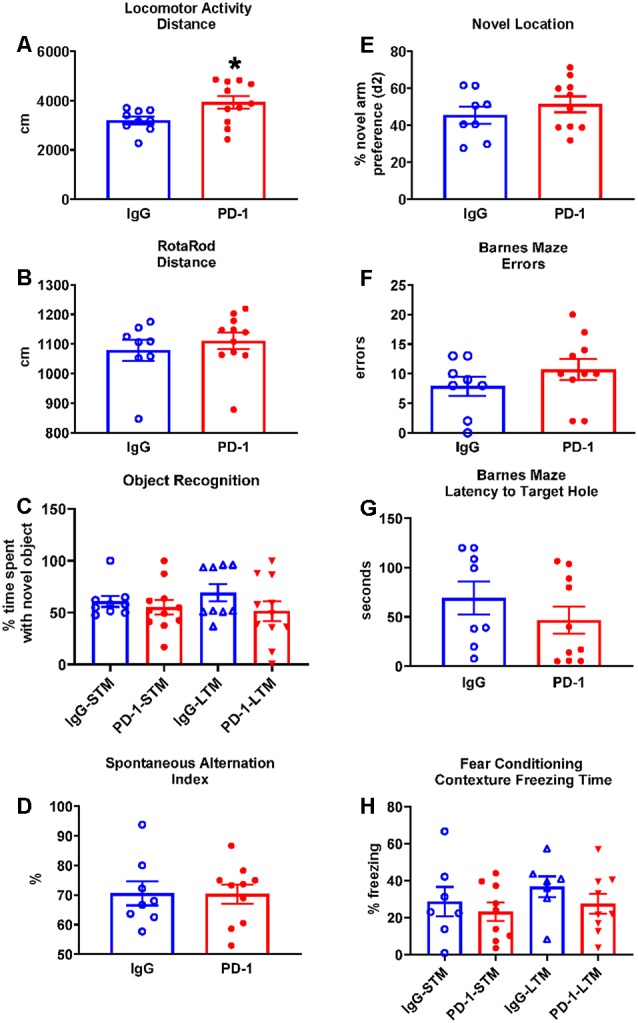
Programmed cell death protein 1 (PD-1) treatment slightly increased locomotor activity but did not improve rotarod performance or affect cognition. **(A)** PD-1 antibody treatment increased distance traveled in a locomotor test compared to the control group. **(B)** PD-1 treatment did not improve rotarod performance compared to the control group. **(C)** PD-1 treatment did not affect memory in an object recognition task. **(D)** PD-1 treatment did not alter spontaneous alternation in the Y-maze or **(E)** preference for the novel arm location in the 2-trial **(F**,**G)** PD-1 treatment did not alter the number of errors or the latency to find the target hole in the Barnes Maze. **(H)** PD-1 treatment did not affect short- or long-term contextual memory in a fear-conditioning task. Each bar represents the group average +/− standard error of the mean (SEM). *p < 0.05. As shown in individual scatterplots, 9–11 PD-1 treated- and 7–9 IgG control mice went through each test. The rotarod results failed normality test and were, therefore, analyzed by the Mann–Whitney test. All the other results passed the normality test and were, therefore, analyzed by unpaired *t*-test.

The PD-1 treatment group was more active in the open field compared to the IgG control group, traveling 23% further (*p* < 0.05; Figure 1A) and at a 23% greater speed {IgG: 3.59 ± 0.18 cm/s, PD-1: 4.43 ± 0.30 cm/s [average ± standard error of the mean (SEM)], *p* < 0.05}.

##### Rotarod

The PD-1 treatment group and the IgG control group did not differ in their performance on the rotarod as measured by the distance traveled (Figure 1B, *p* = 0.35) and the time to fall off the rotating rod (IgG: 107.50 ± 15.70 s, PD-1: 125.10 ± 14.34 s, *p* = 0.42).

#### Cognitive Tests

##### Object Recognition

The PD-1 treatment group did not differ from the IgG control group in the time spent with the novel object in the short-term test (3 h, *p* = 0.33) or the long-term test (24 h, *p* = 0.17; Figure 1C).

##### Spontaneous Alternation

The PD-1 treatment group did not differ from the IgG control group in the spontaneous alternation index (*p* = 0.96; Figure 1D), total arm visits (IgG: 24.13 ± 1.86, PD-1: 29.00 ± 3.32, *p* = 0.25), total distance traveled (IgG: 1576.02 ± 120.98 cm, PD-1: 1870.79 ± 181.09 cm, *p* = 0.22) and total time spent in the arms (IgG: 210.41 ± 8.66 s, PD-1: 211.52 ± 7.73 s, *p* = 0.93).

##### Novel Location

The PD-1 treatment group did not differ from the IgG control group in the preference (d2) for the novel object (*p* = 0.36; Figure 1E), total arm visits (IgG: 9.75 ± 1.74, PD-1: 10.90 ± 2.16, *p* = 0.81), total distance traveled (IgG: 630.83 ± 90.39 cm, PD-1: 779.49 ± 162.41 cm, *p* = 0.70) and the total time spent in the arms (IgG: 130.23 ± 11.12 s, PD-1: 110.55 ± 12.35 s, *p* = 0.27). In trial 1, there was no difference between treatment groups in terms of arm preference (IgG: 58.85 ± 3.61%, PD-1: 57.43 ± 6.25%, *p* = 0.44), total arm visits (IgG: 9.13 ± 0.83, PD-1: 9.50 ± 1.20, *p* = 0.81), total distance traveled (IgG: 560.21 ± 60.49 cm, PD-1: 608.02 ± 69.90 cm, *p* = 0.62) and total time spent in the arms (IgG: 101.95 ± 8.03, PD-1: 90.96 ± 9.88, *p* = 0.42).

##### Barnes Maze

In the probe trial, the PD-1 treatment group did not differ from the IgG control group in the number of errors committed (*p* = 0.41; Figure 1F) or in the latency to reach the target hole (*p* = 0.31; Figure 1G). There were also no differences between groups in the distance traveled to find the platform (IgG: 238.71 ± 51.36 cm, PD-1: 308.82 ± 62.20 cm, *p* = 0.41) or in the average proximity to the target hole during the probe trial (IgG: 68.71 ± 6.11 cm, PD-1: 70.98 ± 7.77 cm, *p* = 0.83).

##### Fear Conditioning

The PD-1 treatment group and the IgG control group did not differ in freezing time when examined under the short-term (*p* = 0.55) and long-term (*p* = 0.27) contextual memory assessment (Figure 1H). There was also no effect of PD-1 treatment on percent freezing time when the white noise cue was on (IgG: 46.10 ± 7.67%, PD-1: 50.59 ± 10.95%, *p* = 0.76) or off (IgG: 15.69 ± 4.61%, PD-1: 22.88 ± 8.30%, *p* = 0.50) in the novel context for the cue conditioning test. In addition, there was no difference between treatment groups in freezing during the initial conditioning session (IgG: 11.45 ± 3.85%, PD-1: 9.98 ± 1.32%, *p* = 0.70).

#### Measurements of Tau Clearance

**Figure 2:**
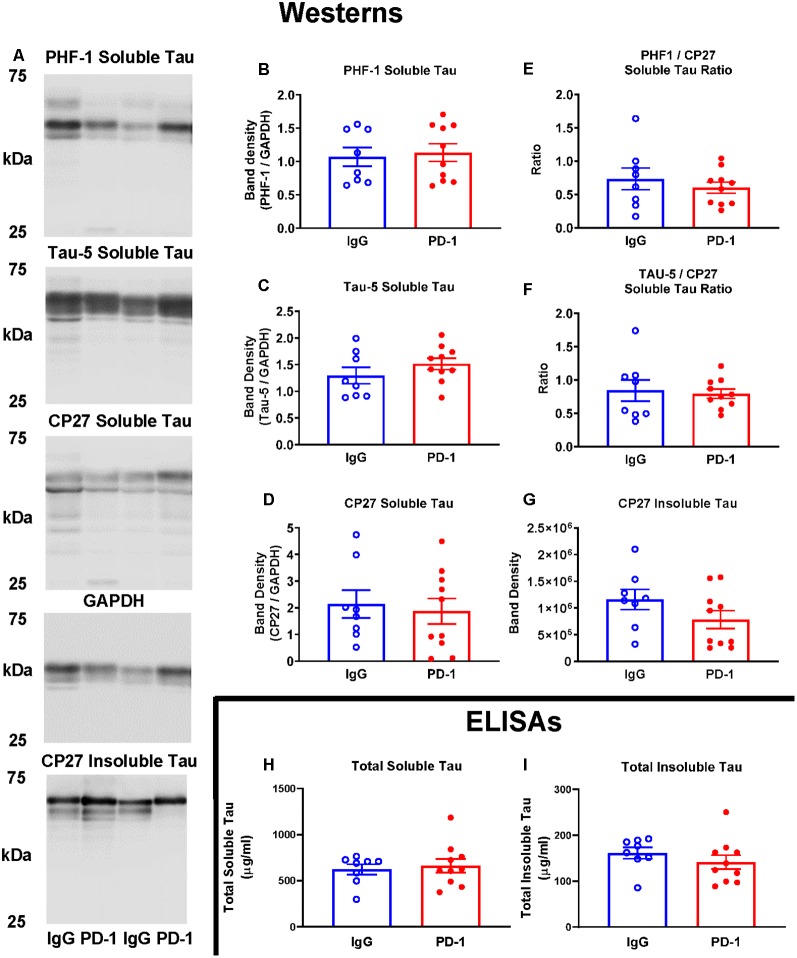
PD-1 treatment did not enhance tau clearance. **(A–F)** PD-1 antibody treatment had no effect on soluble phospho-tau (PHF-1), total tau (Tau-5), total human tau (CP27) or ratios thereof on Western blots of tauopathy mouse brain homogenates, compared to control group. **(A,G)** PD-1 treatment had no effect on insoluble Western blot tau levels in the brain. **(H,I)** Enzyme-linked immunosorbent assay (ELISA) analyses of the same brain homogenates analyzed by Western blots confirmed lack of efficacy of the PD-1 therapy in clearing tau from the brain. Each bar represents the group average +/− SEM. As shown in individual scatterplots, 10 PD-1 treated- and eight control IgG mice were analyzed in each blot or ELISA assay. The total insoluble tau ELISA data failed normality test (IgG group) and was, therefore, analyzed by the Mann–Whitney test. All the other data passed the normality test and were, therefore, analyzed by unpaired *t*-test.

Western blot analysis of soluble and sarkosyl insoluble tau fractions did not reveal any significant differences between the PD-1 treatment group and the IgG control group (Figure 2). Specifically, analysis of the low speed brain homogenate supernatant fraction showed no significant differences in phospho-tau (PHF-1, *p* = 0.74), total tau (Tau-5, *p* = 0.24) total human tau (CP27, *p* = 0.71) or ratios thereof (PHF-1/CP27, *p* = 0.46 and Tau-5/CP27, *p* = 0.78), between the two groups (Figures 2A–F). Likewise, levels of sarkosyl insoluble tau did not differ significantly between the groups (Figures 2A,G, *p* = 0.16).

The lack of effect of PD-1 treatment on tau clearance was confirmed by ELISA measurements of total tau in the soluble (*p* = 0.67) and an insoluble fraction (*p* = 0.23), revealing comparable levels in treatment and control groups (Figures 2H,I).

## Discussion

Several different groups were not able to replicate the original beneficial effects of PD-1 antibody therapy in Aβ plaque models (Baruch et al., 2016; Latta-Mahieu et al., 2018). Although the antibodies and the experimental design, including animal models, were not identical to the pioneering study, a reasonable counter-argument can be made that robust findings under various related experimental conditions are necessary to consider examining this therapeutic approach further in clinical trials.

With regard to the tauopathy study, Swartz and colleagues administered the same PD-1 antibody as in their Aβ model study to tauopathy mice at 8 months of age (a single intraperitoneal injection of 0.5 mg; Rosenzweig et al., 2019). The DM-hTAU mouse model has two tau mutations (K257T and P301S) and has impaired cognition and extensive tau pathology at this age (Rosenzweig et al., 2019). The single antibody injection improved cognition in a T-maze when examined 1 month later in a mixed-sex group. Follow-up analysis of the males revealed similarly improved performance in a Y-maze. Subsequent brain analyses showed a reduction in inflammatory cytokines and diminished tau pathology. Comparable findings were observed with two different PD-L1 antibodies.

To examine the potential of this therapeutic approach, we used the JNPL3 tauopathy mouse model, in which we have previously shown beneficial effects of active and passive tau immunotherapies (Asuni et al., 2007; Boutajangout et al., 2011). The mice received weekly intraperitoneal injections (10 mg/kg or 0.35 mg for a 35 g mouse) for 12 weeks of the same PD-1 antibody and isotype control as used by the Swartz group in their Aβ and tau pathology mice. We used this dosing paradigm in our original passive tau immunotherapy study (Boutajangout et al., 2011), and it was modeled after similar approaches by others in Aβ plaque models, and subsequently by numerous laboratories targeting Aβ, tau or other protein aggregates. This dose is also a typical dose in human trials of Aβ and tau antibodies. Treatment started at 10–11 months of age when this model has already moderate to severe tau pathology, presumably comparable to their DM-hTAU model. If a single injection is effective as per the original study, one would expect 12 weekly injections of a slightly lower dose to be effective as well. Only a minor increase in locomotor activity was observed in the tauopathy mice and no effect in five different cognitive tasks (Object recognition task, Spontaneous alternation task, Novel location task, Barnes maze, and Fear-conditioning task). Furthermore, soluble phosphorylated or non-phosphorylated tau levels or sarkosyl insoluble tau levels were not altered with the prolonged PD-1 antibody therapy as per Western blot analyses, although there was a trend for reduction in soluble PHF1/CP27 tau ratio, and in insoluble CP27 tau. ELISA analyses confirmed a lack of efficacy of the PD-1 antibody therapy on tau clearance.

In addition to the differences in models, the extent of the treatment period, and the number and types of behavioral tests as detailed above, the tau pathology was analyzed differently. Rosensweig primarily analyzed phospho-tau immunoreactivity in the hippocampus (P-Thr212, P-Ser214 and P-Thr231) and aggregated tau in protein extracts, whereas we focused on Western blot analyses of whole-brain homogenate for soluble and insoluble tau and phospho-tau (P-Ser396), followed by ELISA analyses of soluble and insoluble tau. While our approach is more easily quantifiable than histological assessment, it may not detect increased tau clearance within certain brain regions, and there may also be differences in which epitopes are being cleared. However, the efficacy of candidate treatments for clinical development should ideally be sufficiently robust and measurable by any of the standard approaches used to examine the clearance of pathological tau. Considering these negative key findings, it was not warranted to determine if this approach altered cytokine levels and/or led to brain infiltration of monocyte-derived macrophages. Notably, Latta-Mahieu et al. (2018) did not detect macrophage infiltration into the brain in Aβ plaque models following PD-1 treatment.

Cognition has not been extensively examined in the JNPL3 model, presumably because of its motor phenotype, which now is substantially delayed compared to the early days of this model. A prior report indicated that young (5–8.5 months of age) hemizygous JNPL3 mice (*n* = 10) were not impaired as a group in various cognitive tests, compared to non-transgenic littermates (*n* = 11; Arendash et al., 2004). A subsequent study showed normal cognition of hemizygous JNPL3 mice (*n* = 6) in the radial arm water maze and Y-maze at 7–8 months of age, compared to non-transgenic littermates (*n* = 9; Morgan et al., 2008). Similar to these two studies, our JNPL3 mice were of a mixed genetic background, however they were not derived from breeding with the Tg2576 Aβ deposition model. In addition, our mice were homozygous and older at testing (13–15 months of age). In this study, we were not able to compare our JNPL3 mice to non-transgenic mice of the same strain background but based on prior historical data from the Sigurdsson lab and the literature, the JNPL3 mice appear to be impaired in novel object recognition and in the fear conditioning task. In the former test, wild-type mice, younger JNPL3 tauopathy mice, and tauopathy mice in which treatment has been effective, typically spend about 70% of their time with the novel object (Asuni et al., 2007; Boutajangout et al., 2010; Gulinello et al., 2019). In the current study, object preference in both IgG- and PD-1-treated JNPL3 mice did not significantly differ from chance performance (50%). In the latter test, we have previously reported impairments in short- and long-term memory in 12–13 months old JNPL3 mice compared to wild-type mice (Levenga et al., 2013). Similar to our previous work, aged JNPL3 mice exhibited around 35% freezing, which is markedly lower than 50–60% levels of freezing observed in wild-type mice under similar conditions (Levenga et al., 2013; O’Leary et al., 2018).

Various amyloid diseases have over the years been associated with inflammation and an inflammatory stimulus can promote or resolve amyloidosis depending on its strength and various factors in experimental design (Sigurdsson et al., 2002; Lee et al., 2013). It is interesting to note as well that activation of microglia/macrophages is typically associated with increased tau phosphorylation, presumably because of cytokine-mediated activation of cellular kinases (Lee et al., 2013). Accumulation of microglia/macrophages is closely associated with Aβ deposits and these cells are likely involved in plaque clearance. Their link to tau pathology is less clear. With most of the tau lesions being intracellular, the accumulation of phagocytic microglia/macrophages and associated inflammatory signals are much less prominent in pure tauopathies than for Aβ pathology, which is mainly found extracellularly. Hence, linking PD-1 inhibition to Aβ clearance is in many ways feasible, whereas such a mechanistic association with tau clearance is not as apparent. Only a small fraction of tau is found extracellularly (Sigurdsson, 2019). Therefore, it is rather unlikely that acute clearance of extracellular tau within a 1 month period following a single antibody injection could have an extensive effect on intracellular tau aggregates. In addition, any indirect effect of PD-1 blockade on intracellular tau is more likely to promote tau hyperphosphorylation based on prior studies as mentioned above (Lee et al., 2013). If the effect is mainly extracellular, a prolonged treatment such as ours, with weekly antibody injections for 12 weeks, would be more likely to eventually lead to removal of intraneuronal tau aggregates. However, that was not the case in our study.

Finally, it can be argued that the effects of PD-1 blockade on tau pathology and associated behavioral impairments may differ between acute vs. chronic treatment. Although possible, prolonged treatment will be necessary to effectively treat chronic slowly progressing diseases like Alzheimer’s disease and other tauopathies. Unfortunately, chronic treatment with a PD-1 blocking antibody, as could be envisioned to be applied clinically, did not clear tau pathology or substantially improve behavior under our experimental conditions. Therefore, further research is needed before clinical trials based on PD-1 checkpoint immune blockage are devised for tauopathies.

## Data Availability Statement

The datasets generated for this study are available on request to the corresponding author.

## Ethics Statement

The animal study was reviewed and approved by New York University School of Medicine, IACUC committee.

## Author Contributions

HR took care of the mouse colony and immunized the mice. YL performed the behavioral studies with help from BG-L and AM who designed some of the tests. YL performed the western blots and related analyses. LS-B designed and performed the ELISAs. All the authors contributed to the method section. ES designed the experiment and wrote the article. YL and AM edited the article.

## Conflict of Interest

ES is an inventor on various patents and patent applications that are assigned to New York University. Some of the patents on tau immunotherapies and related diagnostics are licensed to and are being co-developed with H. Lundbeck A/S.

The remaining authors declare that the research was conducted in the absence of any commercial or financial relationships that could be construed as a potential conflict of interest.
